# Triangular Black Phosphorus Atomic Layers by Liquid Exfoliation

**DOI:** 10.1038/srep23736

**Published:** 2016-03-30

**Authors:** Soonjoo Seo, Hyun Uk Lee, Soon Chang Lee, Yooseok Kim, Hyeran Kim, Junhyeok Bang, Jonghan Won, Youngjun Kim, Byoungnam Park, Jouhahn Lee

**Affiliations:** 1 Advanced Nano-surface Research Group, Korea Basic Science Institute, Daejeon, 34133, Republic of Korea; 2 Department of Applied Chemistry and Biological Engineering, Chungnam National University, Daejeon, 34134, Republic of Korea; 3 Spin Engineering Physics Team, Korea Basic Science Institute, Daejeon, 34133, Republic of Korea; 4 Department of Materials Science and Engineering, Hongik University, Seoul, 04066, Republic of Korea

## Abstract

Few-layer black phosphorus (BP) is the most promising material among the two-dimensional materials due to its layered structure and the excellent semiconductor properties. Currently, thin BP atomic layers are obtained mostly by mechanical exfoliation of bulk BP, which limits applications in thin-film based electronics due to a scaling process. Here we report highly crystalline few-layer black phosphorus thin films produced by liquid exfoliation. We demonstrate that the liquid-exfoliated BP forms a triangular crystalline structure on SiO_2_/Si (001) and amorphous carbon. The highly crystalline BP layers are faceted with a preferred orientation of the (010) plane on the sharp edge, which is an energetically most favorable facet according to the density functional theory calculations. Our results can be useful in understanding the triangular BP structure for large-area applications in electronic devices using two-dimensional materials. The sensitivity and selectivity of liquid-exfoliated BP to gas vapor demonstrate great potential for practical applications as sensors.

The increasing demand for elemental 2D materials, such as graphene and transition metal dichalcogenides (TMDCs), emerged for applications in sensors^1^,^2^,^3^, optoelectronics^4^,^5^, flexible displays^6^, and photovoltaics^7^,^8^. Although graphene has high carrier mobility^9^, its zero band gap limits various electronic applications^10^. Recent studies found that phosphorene, monolayer of black phosphorus, can be mechanically exfoliated similarly to graphene^10^,^11^,^12^,^13^,^14^. Contrary to graphene, black phosphorus (BP) has a band gap of 0.3–2 eV, which allows it to be exploited as an excellent semiconductor and potentially an alternative for graphene^7^,^10^,^15^,^16^,^17^,^18^. In addition, we reported that BP can be applied to produce highly efficient photocatalyst^19^,^20^.

Previous studies on the electronic applications of BP^21^,^22^ are based on the layer-dependent nature of BP^23^,^24^. Bulk BP layers are stacked together by the van der Waals force and can be easily peeled off layer by layer^25^. Most of the recent studies on BP are based on mechanically exfoliated BP bulk samples^24^. Although mechanical exfoliation is an effective technique to cleave bulk materials into mono or few layers, it is limited by a scaling process. Therefore, it is necessary to employ a liquid method for large scale and various practical applications.

Previous work proved that BP deposited on SiO_2_/Si by the liquid exfoliation forms high-quality single crystalline nanoflakes^26^. Brent *et al.* reported that few-layer phosphorene was produced by liquid exfoliation of BP in *N*-methyl-2-pyrrolidone^27^. Other studies on BP layers by liquid exfoliation showed that BP atomic layers look like debris or flakes with a random shape or orientation^28^,^29^,^30^. None of them, however, showed well-defined crystalline thin films covering whole substrates. Hence, they appear to be inappropriate for thin-film based electronic applications.

Our liquid exfoliation method uses higher sonication energies than the conventional method and different solutions, as illustrated in Fig. 1a, which allows us to obtain highly crystalline triangle-shaped BP thin films. Unlike the liquid-exfoliated BP flakes with random shapes reported in the literature, our BP sample cleaves into faceted triangular atomic layers, similar to triangle-shaped flakes commonly observed in MoS_2_ nanocrystals^31^,^32^,^33^,^34^. The thickness and the density of BP layers on the substrate can be easily changed by adjusting the sonic energy and the concentration of BP in our sample. Using our method, it is possible to produce ultrathin BP films varying in thickness from monolayers to few layers. It can be challenging to design uniform thin films using BP flakes with random shapes because high coverage of BP is needed to uniformly cover the whole substrate, which results in thick stacked BP layers close to bulk BP. The fabrication of nano devices using single BP flakes has been demonstrated in the literature^35^,^36^,^37^, which is not suitable for large-area processing. On the other hand, triangular BP layers can effectively cover the whole substrate with low coverage, which facilitates the fabrication of uniform thin films. This makes the liquid-exfoliated BP a perfect candidate for large-area applications in electronic devices.

In this study, we used ultrasonic energy (Fig. 1b) to exfoliate bulk BP, breaking it down into mono or few layers, and investigated the surface properties of liquid-exfoliated BP multilayers on SiO_2_/Si (001) substrates using atomic force microscopy (AFM), scanning electron microscopy (SEM), transmission electron microscopy (TEM), Raman spectroscopy, and X-ray diffractometer (XRD) in combination with density functional theory (DFT) calculations. We found that the liquid-exfoliated BP forms a layer-by-layer structure with well-defined triangle-shaped crystals. In addition, the practical applications of liquid-exfoliated BP as chemical sensors were explored using field-effect transistors (FETs) and diode devices.

## Results

### Crystal structure of BP

**Figure 1:**
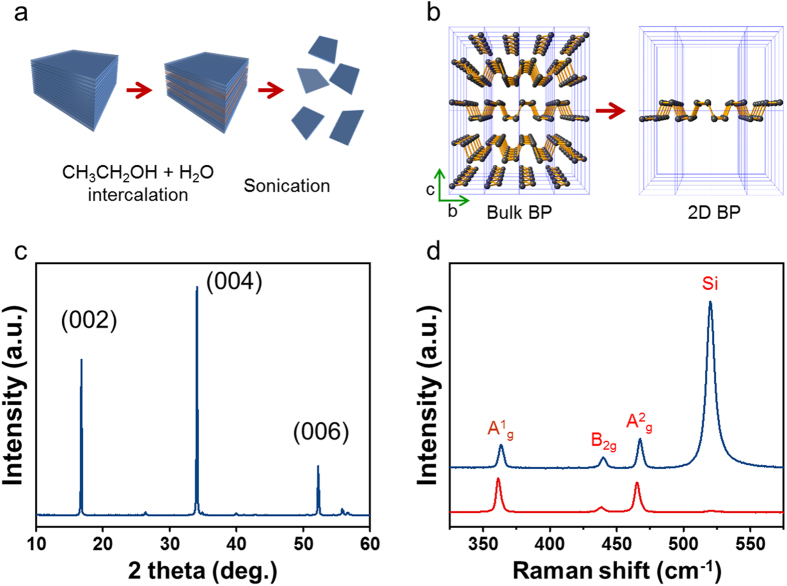
(**a**) Schematic illustration of liquid exfoliation procedure of BP using high energy ultrasonic irradiation (**b**) The corresponding atomic modelling depicting bulk BP cleaved into monolayer BP. (**c**) XRD pattern of liquid-exfoliated BP indicating the crystalline phase of BP. (**d**) Raman spectra of liquid-exfoliated BP. Inset: three types of optical phonon modes of BP depicting A^1^_g_ at 363 cm^−1^, B_2g_ at 440 cm^−1^ and A^2^_g_ at 467 cm^−1^.

The XRD measurements were carried out to examine the crystal structure of BP samples produced by liquid exfoliation. As shown in Fig. 1c, sharp and strong peaks were observed at 2θ = 16.89° for the (002) plane, 34.17° for the (004) plane and 52.32° for the (006) crystal plane. Based on the XRD data, we found that our BP samples have an orthorhombic structure with lattice constants of a = 3.32 Å, b = 4.38 Å and c = 10.48 Å, which agrees with the previous studies for BP^38^.

Raman spectroscopy measurements were performed to verify the crystalline structure of liquid-exfoliated BP. BP flakes were obtained from a droplet of the liquid- exfoliated BP on SiO_2_/Si (001). The sample was dried to remove the solvent. Figure 1d shows the Raman spectra measured at two different positions within a single BP flake. In the spectra, there are four prominent peaks at 363 cm^−1^, 440 cm^−1^, 467 cm^−1^ and 520 cm^−1^. The BP flake is thin enough to observe the Si peak at 520 cm^−1^ [ref. 39]. The peaks at 363 cm^−1^, 440 cm^−1^ and 467 cm^−1^ are known to result from the optical phonons of a crystalline lattice of BP^40^. They match the Raman shifts attributed to one out-of-plane vibration mode (A^1^_g_) at 363 cm^−1^ and two in-plane vibration modes (B_2g_ and A^2^_g_) at 440 cm^−1^ and 467 cm^−1^. As shown in the inset of Fig. 1d, BP atoms vibrate along the *c* axis represented as A^1^_g_ and along the *b* axis indicated as A^2^_g_. The model for B_2g_ illustrates the in-plane vibration mode of BP atoms. The peak for each vibration mode of our BP sample deviates from that of bulk BP in the literature: 365 cm^−1^ for A^1^_g_, 442 cm^−1^ for B_2g_, and 470 cm^−1^ for A^2^_g_^39^. Instead, the positions of these peaks are consistent with the Raman shift values of BP multilayers by Lu *et al.*^41^, which implies that BP prepared by liquid exfoliation exhibits the crystalline thin-film phase.

### Surface topography of BP

**Figure 2:**
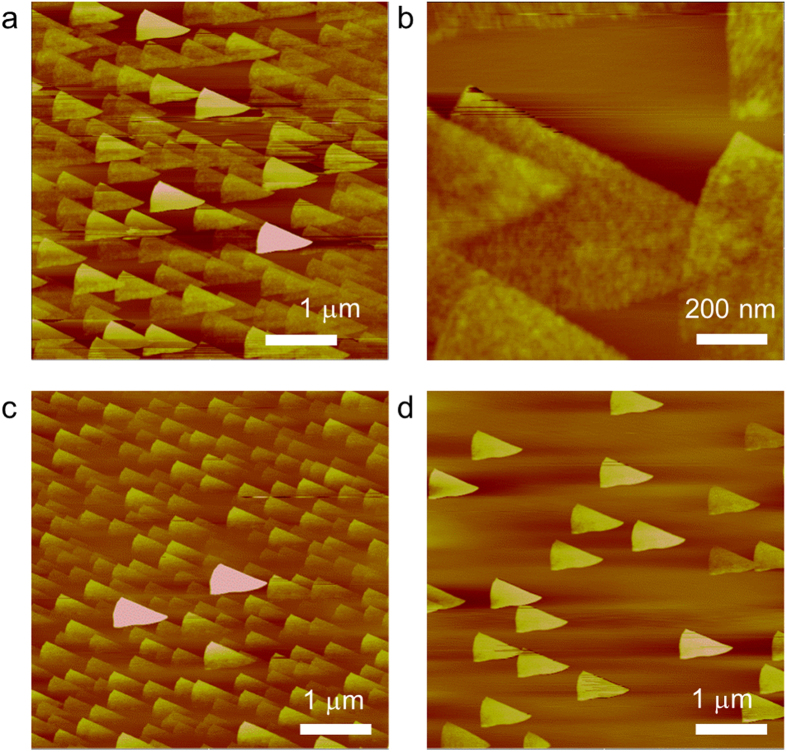
Structure of liquid-exfoliated BP demonstrating single crystalline thin film phase. (**a**) AFM image of triangle-shaped BP layers formed on SiO_2_/Si (001) spin-coated at 2500 rpm (5 μm × 5 μm), (**b**) Enlarged AFM image of BP layers (790 nm × 790 nm) (**c**) Degradation of BP thin film with lower concentration (5 μm × 5 μm). (**d**) Degradation of BP thin film with higher concentration (5 μm × 5 μm).

The surface morphology of BP was investigated using AFM. The repeatable measurements from a number of BP samples demonstrate that BP forms triangle-shaped atomic layers on SiO_2_/Si (001). As shown in Fig. 2, the triangular BP layers are faceted, which proves that the BP layers are single crystalline. The different contrast in the images indicates that BP forms a layered structure on SiO_2_/Si (001), as shown in Fig. 2a. The height of the triangular BP crystal in Fig. 2b is approximately 1 nm. A series of height measurements revealed that the triangular BP crystals in Fig. 2a have three different thicknesses: 1.10 ± 0.16 nm, 1.71 ± 0.12 nm and 2.50 ± 0.2 nm. The height data indicate that there are at least two BP atomic layers in each 1 nm-high triangle and roughly 4–8 atomic layers stacked on the higher triangles. The average lateral size of the triangular BP crystals is approximately 756 ± 25 nm. We also experimented with various conditions such as different sonication time and different spin coating rpm. In varying the sonication time from 5 min. to 40 min., we found that a change in the sonication time resulted in a change in the concentration of BP in the solution, which leads to a change in the size, density, and thickness of BP crystals. The thinnest BP layer we observed was about 3 Å in height.

Figure 2c,d show the AFM images of liquid-exfoliated BP samples with two different concentrations. The concentration of the BP sample in Fig. 2d is higher than that of Fig. 2c. The biggest difference is that the number of thicker BP islands (>8 nm high) is larger in Fig. 2d. This means the higher BP concentration results in the larger density of higher triangles. The AFM image in Fig. 2c shows that the bottom part of each triangular crystal is not as clear as the top part because the BP thin film started to degrade (see Supplementary Fig. S2). The reason why the number of triangular BP crystals is less in Fig. 2d is that the AFM image was taken a few days after the sample was prepared. The thin BP islands (~1 nm high) were gone by that time and only thick BP crystals were survived.

**Figure 3:**
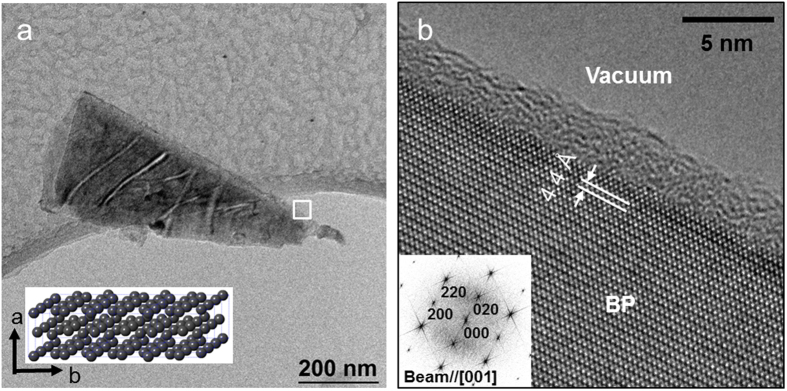
(**a**) Bright-field TEM image of liquid exfoliated BP on a quantifoil grid. Inset: BP atomic model (**b**) High-resolution TEM image at atomic scale. Inset: fast Fourier transform.

The formation of triangular BP layers is a characteristic feature of a BP crystal regardless of the substrates. Figure 3a shows a bright-field TEM image of BP produced by liquid-exfoliation formed on an amorphous carbon grid. The TEM image shows a BP flake formed in a triangular shape, which coincides with the AFM image. This morphological feature allows easy identification of the crystal orientation. The size of the crystal is approximately 800 nm, which is consistent with the size of the BP triangle in the AFM image. The atomic-scale image in Fig. 3b shows the lattice structure of BP from the straight edge of the triangular BP crystal marked as a rectangular box in Fig. 3a and the corresponding fast Fourier transform (FFT) pattern. The interlayer distance measured from the high-resolution TEM image was approximately 4.4 Å along the *b*-axis of the unit cell. This interlayer distance is very close to the lattice constant *b* = 4.38 Å measured from the XRD pattern. The comparison between the atomic model in the inset in Fig. 3a and the FFT pattern in Fig. 3b suggests that the straight edge of the triangular BP crystal is assigned to the (010) plane. The only difference between TEM and AFM results is that the BP flake in the TEM image is thicker than those of AFM images. Thin BP flakes degraded fast due to the incident electron beam during the TEM measurements.

We also carried out SEM measurements in order to verify the observations from AFM. Similar to the TEM experiment, thin BP layers were hard to image during the SEM measurements because of the electron beam damage or a contamination problem (see Supplementary Fig. S2).

### Practical Applications as Sensors

**Figure 4:**
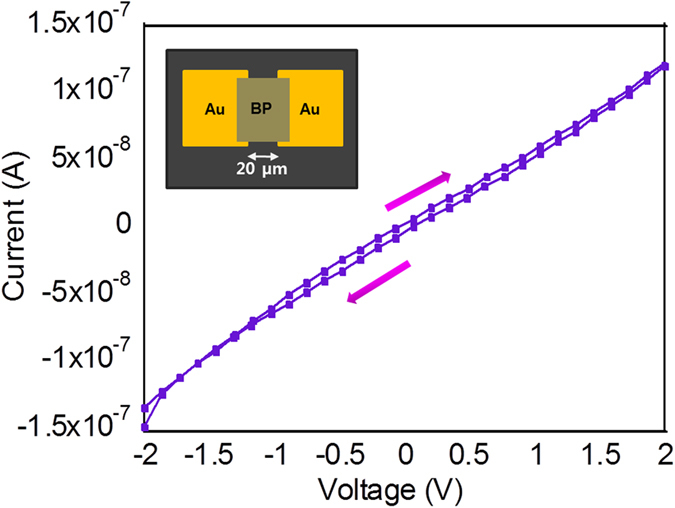
Current-voltage characteristic curve of liquid- exfoliated BP drop-casted on an FET with a channel length of 20 μm.

The carrier transport properties of liquid exfoliated BP were examined using a bottom-contact FET. The liquid-exfoliated BP was drop-casted on an FET where the source and the drain electrodes are pre-patterned onto the SiO_2_ gate dielectric substrate. The inset in Fig. 4 shows the structure of a BP FET with the channel length of 20 μm. Our results revealed that the drain current modulation induced by the gate electric field was not observed (see Supplementary Fig. S4a), which is attributed to the degradation of the FET device resulted from surface defects, oxidation and interface charge traps^42^ since ultrathin BP FETs are more sensitive to interface states than bulk BP. Instead the current-voltage (I–V) response of the BP FET was obtained as shown in Fig. 4. The arrows indicate the sweeping directions. The voltage was swept from −2 V to 2 V and the measured current was in the order of 10^−7^ A.

We tested if liquid-exfoliated BP can be applied in other electrical devices. The sensitivity of BP to the surrounding atmosphere can be applied as vapor sensors. Previous study reported that gas molecule adsorption led to change in the charge transport in phosphorene, which implies that phosphorene can be used as superior gas sensors^43^. We fabricated liquid-exfoliated BP sensors and examined the current as a function of time before and after the exposure to acetone (C_3_H_6_O). Acetone is a highly flammable toxic chemical widely used to dissolve substances, to make plastics, and to clean products. We found that the current was increased by two orders of magnitude after the device was exposed to acetone (see Supplementary Fig. S4b). This implies that liquid-exfoliated BP selectively detects acetone vapor and the sensing capability of BP can be used for practical applications.

### Theoretical calculations

**Figure 5:**
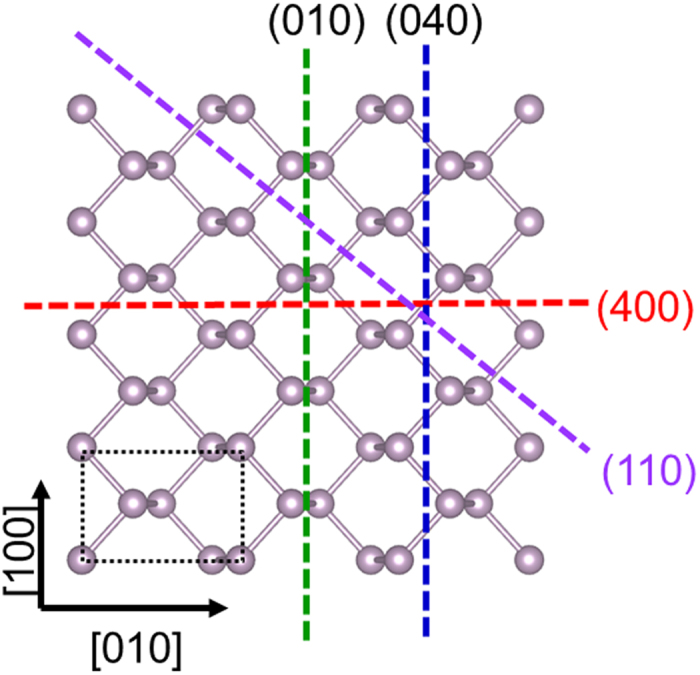
Atomic structure of BP monolayer calculated by DFT. The green, blue, purple and red dashed lines represent (010), (040), (400) and (110) planes, respectively. The figure was generated by the VESTA software.

We performed DFT calculations to investigate the energetically preferred edge of the BP layer. Figure 5 shows the atomic structure of BP monolayer, and the black dotted box represents the unit cell. Using one-dimensional BP nanoribbons where the atomic layer is cut along the different lines as shown in Fig. 5, the formation energy of the edges was calculated as follows


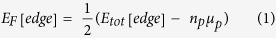


where *E*_*tot*_[*edge*] is the total energy of the edge system, *n*_*p*_ is the number and *μ*_*p*_ is the chemical potential of phosphorus atoms. The factor 1/2 represents two equivalent edges in the nanoribbon. *μ*_*p*_ was calculated from the total energy of the pristine BP monolayer. The formation energies per unit cell for the (400) and (040) edges are the same as 1.24 eV (see Supplementary Table S1). On the contrary, the formation energy per unit cell for the (010) edge is 0.69 eV. Similarly, the formation energies per unit length for the (040), (400), (110) and (010) edges are 0.37, 0.27, 0.34 and 0.21 eV/Å, respectively, which indicates that the (010) edge is energetically most stable.

**Figure 6:**
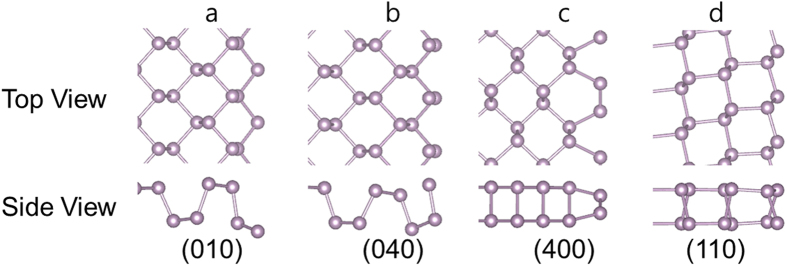
Top and side views of atomic structure of BP monolayer calculated by DFT. (**a**) (010) plane, (**b**) (040) plane, (400) plane and (**d**) (110) plane.

Figure 6 depicts the atomic model of the top view and the side view of the BP monolayer in DFT calculations. All the edges undergo significant lattice relaxations, except the (110) edge. The edge reconstructions result in a decrease in the energy of the edges by lowering or self-passivating dangling bond levels. We calculated the formation energy of the (110) edges and found that the (110) edge also has higher energy density than the (010) edge (see Supplementary Table S1). While the (040) and (400) edges have two dangling bonds per unit cell, the (010) edge has only one dangling bond, which explains why the (010) edge has the lowest energy. Our first-principle calculations show that the (010) edge is the most stable structure. Comparison between the AFM and TEM images, as well as the calculations, suggests that the straight edge of each triangular BP layer is formed along the (010) plane because it is an energetically preferred facet.

## Discussion

Our XRD and Raman data clearly indicate that the liquid-exfoliated BP has a single crystalline phase. The AFM results show that it is possible to fabricate BP thin films with triangle-shaped crystals in various thicknesses from monolayers to multilayers of BP. In the AFM images of BP in Fig. 2, notice that the lower side of each triangle is curved while the upper side is straight, which presumably might reflect different edge reconstructions^15^. Previous study of DFT calculations on BP predicted that 2D phosphorene has two edge states: armchair and zigzag^15^. Our ongoing work to differentiate armchair edges from zigzag edges in the triangular BP layer is in progress.

The AFM results show that the liquid-exfoliated BP samples degrade with time as shown in Fig. 2 and Fig. S2 (see Supplementary Fig. S2). The reason why there are no thin BP layers observed in Fig. 2d is that over time thinner BP layers disappear faster than thicker layers. Previous studies reported that BP atomic layers degrade in ambient air, which arises from surface reactions with ambient gas molecules. In addition, the degradation of BP is ascribed to surface defects, oxidation and interface trap states^42^. The degradation process of BP has never been observed in real-space images. According to the AFM images, the bottom part of each triangular BP crystal disappears first, and the straight edge part degrades more slowly (see Supplementary Fig. S2). We assume that the smearing phenomenon may result from different edge reconstructions of a BP island, and the bottom part of each island may have an atomic structure that breaks up easily in ambient air^23^. There might be more defects leading to surface oxidation in the bottom part of the triangular BP crystals. Atomic-scale study is required to examine the edge terminations.

The structure of liquid-exfoliated BP was examined based on the surface topography in combination with DFT calculations. Our results demonstrated that BP produced by the liquid exfoliation forms triangular crystalline structure on SiO_2_/Si (001) as well as amorphous carbon substrates. The triangular BP crystals are faceted with the preferred orientation of (010) plane, which suggests that liquid-exfoliated BP forms a well-defined thin-film phase on the substrates. In addition, varying the concentration of BP in the solution can change the size, thickness, and density of the BP crystals.

Our findings show that simple fabrication for large area processing and the layer-by-layer structure of highly crystalline BP films allow for integration into electronic devices. Particularly, BP can be optimized for applications in chemical sensors since the layer-by-layer structure of the BP layer induces uniform reaction sites. Indeed, our chemical BP sensors demonstrated that the highly crystalline faceted structure of BP layers has great potential for high selectivity and sensitivity of chemical vapor (see Supplementary Figs S4 and S5). Furthermore, facile processing conditions into hetero-structured thin films provide flexibility in designing functional interfaces requiring efficient charge transfer, which enables applications as photovoltaic and hydrogen producing devices.

## Methods

### Experimental

Structural studies of the BP on SiO_2_/Si (001) were performed using AFM (Digital Instruments: Nanoscope Multimode IV). The AFM measurements were carried out in four different areas of each sample with different scan sizes from 1 μm to 15 μm. Antimony-doped *n*-type Si (001) with 0.008–0.02 Ω·cm resistivity was used as a substrate and cleaned using the interuniversity microelectronics center (IMEC) process^44^. The bulk BP was cleaved and chemically exfoliated into few atomic layers of BP in an appropriate solution. Figure 1(a) depicts the liquid-exfoliation procedure of BP. The 0.2 g of BP 6.4 mmol was dispersed in a 200 mL of deionized water (100 ml)/anhydrous ethyl alcohol (100 ml) solution by high-energy ultrasonic irradiation for 20 min. to form few layers of BP. The dispersion of BP layers was performed using high-energy ultrasound at 20 kHz applied from the top of a polypropylene bottle reactor (500 mL) using a Sonics and Materials VC750 ultrasonic generator. The electrical energy input was maintained at 100 W. As shown in Fig. 1b, once bulk BP is cleaved into monolayers, a phosphorus atom is covalently bonded with three neighboring phosphorus atoms to form a puckered Honeycomb structure in a 2D sheet^25^,^45^. The crystalline structure of BP was investigated using XRD (Rigaku RDA-cA X-ray diffractometer) patterns obtained using Cu Kα radiation through a nickel filter. Raman spectroscopy was also performed (Renishaw, RM1000-Invia) in a backscattering configuration excited with a visible laser light (wavelength = 514 nm), a notch filter cut-off frequency of 50 cm^−1^, and a focus spot size of 5 μm. Spectra were collected through a 100× objective and recorded with 1800 lines mm^−1^ grating providing the spectral resolution of ~1 cm^−1^. To avoid laser-induced heating and ablation of the samples, all spectra were recorded at low power levels P ~ 0.1 mW and short integration times (~5 s). For structural analysis using AFM, liquid-exfoliated BP in a solution was spin-coated at 2500 rpm for 30 s twice and baked in an oven overnight. For TEM measurements, the BP sample was dispersed on a qantifoil TEM grid and dried thoroughly. Then the measurements were carried out using a Libra 200 energy-filtered TEM with an aberration corrector (Carl Zeiss). For SEM measurements, spin coated BP on a Si substrate was investigated by means of MERLIN (Carl Zeiss), with the accelerating voltage ranging from 100 eV to 1 kV for BP films. A bottom-contact FET was fabricated in which source and drain electrodes (Au/Ti) were patterned on a 200 nm thick SiO_2_ gate dielectric using photolithography. Highly doped *p*-type silicon was used as a gate electrode. BP thin films were drop-casted from a solution and dried at 90 °C in air. All the electrical measurements were carried out in air.

### Theoretical

Our calculations are based on DFT with the Perdew-Burke-Ernzerhof exchange-correlation functional^46^, as implemented in the VASP code^47^. Projector augmented wave potentials are used to represent ion cores^48^. Wave functions are in a plane-wave basis with an energy cut-off of 400 eV. Optimized lattice constants were used, and all atoms were fully relaxed until the residual forces are less than 0.01 eV/Å. The Brillouin zone is sampled by k-point meshes equivalent to the 15 × 11 Monkhorst-Pack mesh for a (1 × 1) cell. BP is separated from its periodic images by 12 Å vacuum regions.

## Additional Information

**How to cite this article**: Seo, S. *et al.* Triangular Black Phosphorus Atomic Layers by Liquid Exfoliation. *Sci. Rep.* **6**, 23736; doi: 10.1038/srep23736 (2016).

## Supplementary Material

Supplementary Information
