# Supplementary material for: Triangular Black Phosphorus Atomic Layers by Liquid Exfoliation
Source: Sci Rep. 2016 Mar 30;6:23736. doi: 10.1038/srep23736 (PMC4812320; doi:10.1038/srep23736)
Supplement: Supplementary Information [file srep23736-s1.pdf]

Supplementary Information

Triangular Black Phosphorus Atomic Layers by Liquid Exfoliation

Soonjoo Seo[1], Hyun Uk Lee[1*], Soon Chang Lee[2], Yooseok Kim[1], Hyeran Kim[1],Junhyeok Bang[3], Jonghan Won[1], Youngjun Kim [4], Byoungnam Park[4] and Jouhahn Lee[1*]

[1]Advanced Nano-surface Research Group, Korea Basic Science Institute, Daejeon, Republic of Korea

[2]Department of Applied Chemistry and Biological Engineering, Chungnam National University, Daejeon 305-764, Republic of Korea

[3]Spin Engineering Physics Team, Korea Basic Science Institute, Daejeon, Republic of Korea

[4]Department of Materials Science and Engineering, Hongik University, Seoul, 04066, Republic of Korea

Electronic mail:leeho@kbsi.re.kr, jouhahn@kbsi.re.kr

Contents:

1. **Edge stability of black phosphorus (BP) monolayer**
2. **Reliability of the atomic force microscopy (AFM) results**
3. **Aging effect of chemically exfoliated black phosphorus**
4. **Practical applications as chemical sensors**

1. Edge stability of black phosphorus (BP) monolayer

Table S1 shows the calculated formation energies of BP for four different BP edges. As shown in the table, the formation energies per unit cell for (040) and (400) planes are the same; on the contrary, the formation energy for the edge along the (010) plane is much lower. Likewise, the energy density for the (010) edge is the lowest, which suggests that the edge along the (010) edge is energetically the most stable structure in the BP monolayer. The (400) and (040) edges have two dangling bonds per unit cell while the (010) edge has only one dangling bond, which describes why the (010) edge has the lowest formation energy.

Edges	Edge formation energy	
	Unit cell (eV)	Density (eV/Å)
(010)	0.69	0.21
(040)	1.24	0.37
(400)	1.24	0.27
(110)	-	0.34

Table S1. Edge formation energy. The energies are shown in two different units: formation energy per unit cell and per unit length (energy density).

2. Reliability of the AFM results

Fig. S1 shows the AFM images taken in four different areas within the same BP sample. Triangle-shaped BP layers were observed all over the sample. Each spot was scanned repeatedly with different scan conditions using different tips, and the results were consistent. The effect of AFM tips, including double tips or tip artifacts, can be ruled out by the reproducibility and the consistency of the data.

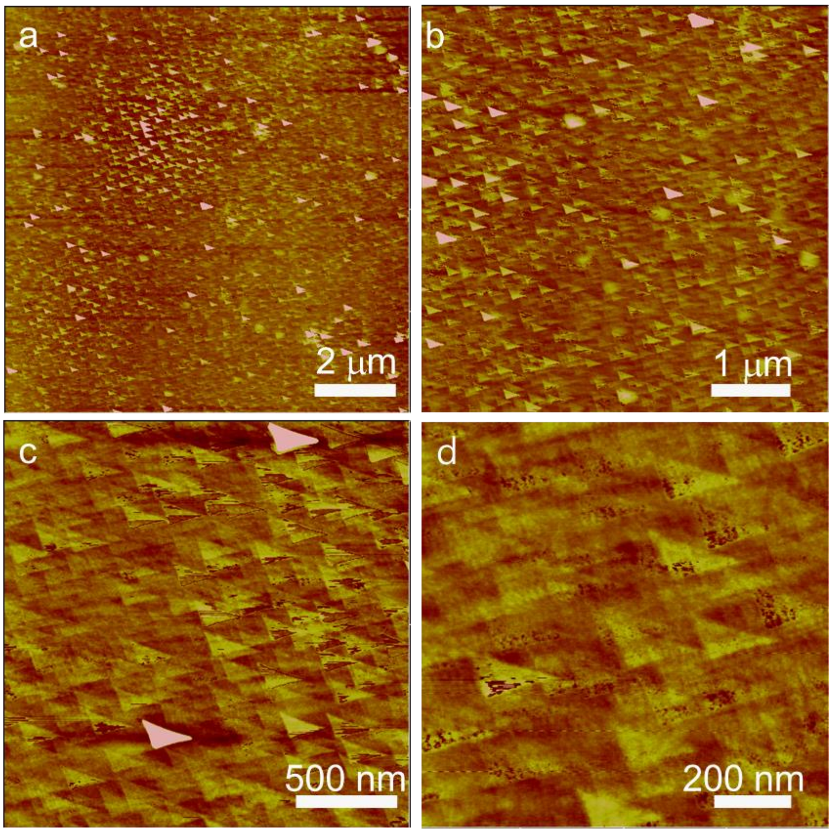

Fig. S1. AFM images obtained in different areas within the same BP sample spin-coated at 2000 rpm on SiO_2/ Si (001). The scan sizes are (a) 10 μm × 10 μm (b) 5 μm × 5 μm (c) 2 μm × 2 μm (d) 1 μm × 1 μm.

3. **Aging Effect of liquid exfoliated black phosphorus**

Our data proved that the liquid-exfoliated BP degrades with time. AFM measurements were carried out to investigate the aging effect of BP five days after BP was spin-coated on the SiO_2/Si (001) substrate. As shown in the AFM image in Fig. S2, the bottom part of each triangular BP crystal disappeared. This means the atomic structure of the curved edge of triangular BP layers tends to break apart easily. In addition, thinner BP layers degrade faster than thicker BP layers.

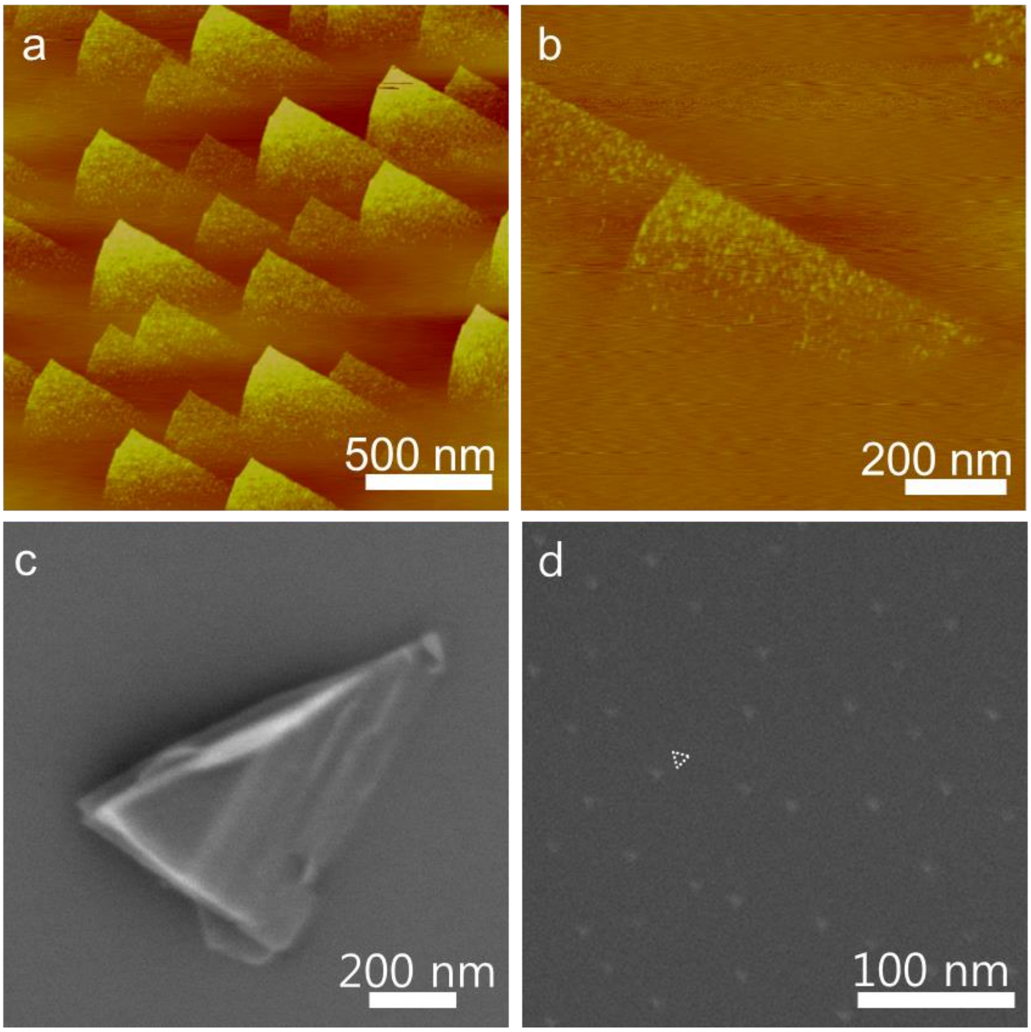

Fig. S2. AFM images of chemically exfoliated BP showing an aging effect. Image sizes: (a) 2 μm × 2 μm and (b) 1 μm × 1 μm. (c) SEM image of a liquid exfoliated BP crystal. (d) SEM image of liquid exfoliated triangular BP islands.

We also performed scanning electron microscopy (SEM) measurements on liquid-exfoliated BP spin-coated on SiO_2/Si (001) with accelerating voltages from 100 V to 1 kV. Although we used low accelerating voltages, the sample damage was so severe that the BP layers disappeared quickly. Thus, it was difficult to obtain SEM images of BP thin films prepared by liquid exfoliation. Thick BP crystals depicted as the middle step in Fig. 1a are relatively easier to image by SEM. Fig. S2 (c) shows a thick triangle-shaped BP crystal, which can easily cleave into mono or few layers of BP with more sonication energies. However, thin BP layers, the same as those observed in the AFM images, were not visible after the exposure to the electron beam during SEM measurements, which makes it impossible to obtain high-resolution SEM images of few-layer BP. Only captured SEM images of BP thin films were acquired after a quick scan. Fig. S2 (d) shows small-sized BP crystals formed on SiO_2/Si (001) which are smaller than those of AFM images. The size of the triangle can be changed by adjusting the sonication time and the power. The resolution of the SEM image is not great, but the triangular BP crystal marked as a dotted triangle in Fig. S2 (d) were observed all over the sample and they were gone after a few scans, which might be attributed to the decomposition of the samples by the electron beam or contamination, which makes it even harder to try high-resolution SEM imaging and energy dispersive x-ray spectroscopy (EDS) mapping.

In order to investigate the aging effect of BP by beam damage, Raman measurements were also carried out on mechanically exfoliated bulk BP flake on SiO_2/Si (001). Fig. S3 (a) shows the Raman spectra of the bulk BP sample exposed to a laser for 300 min. The Raman spectra clearly show that the intensity of the Si peak decreases with increasing time. Unlike the optical image (left) in Fig. S3 (b), the optical micrograph (right) of the BP sample shined with the laser for 300 min. shows a dark spot, which implies that the increase in the exposure

time to the laser resulted in the defect in the BP sample. The possible origin of the defect on the BP layer may be overheating and etching by the laser beam. Considering that the peak intensity of the normalized Si increases with increasing time of laser exposure (Fig. S3 (c)), the BP sample degraded due to the laser beam damage. This also explains the difficulties in imaging BP thin films due to electron beam damage during SEM measurements.

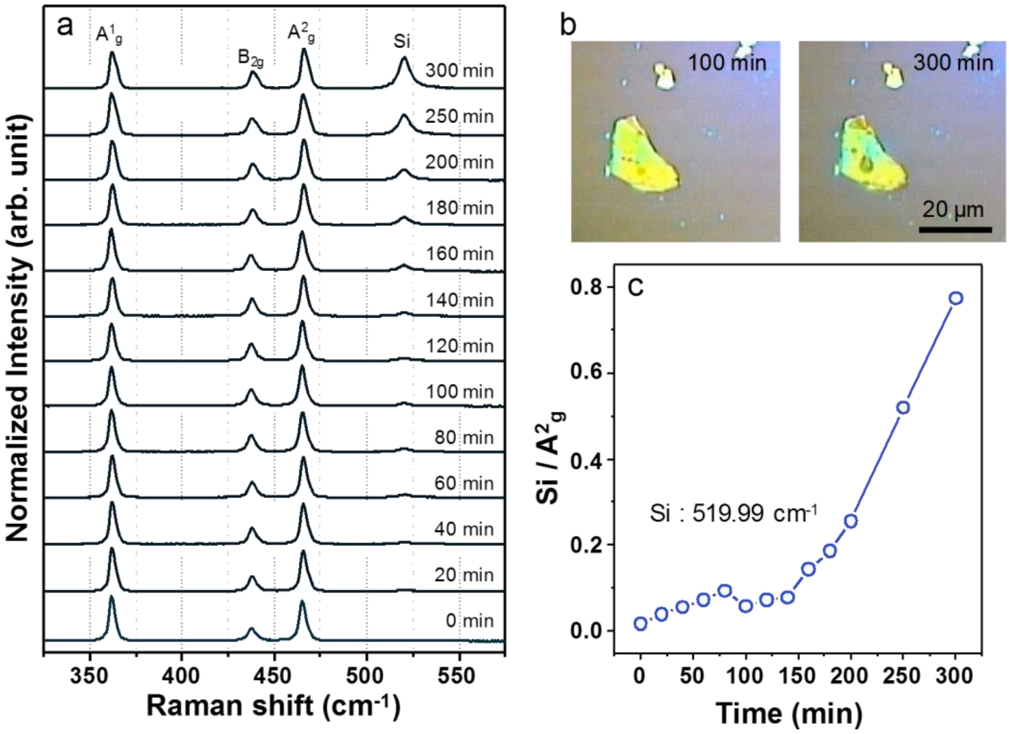

Fig. S3. Raman spectra of mechanically exfoliated bulk BP flake on silicon. (a) Raman spectra measured in air with a 472.8 nm excitation laser in same position of a BP flake with laser exposure time from 10 min to 300 min. In the spectra there are four prominent peaks at ~361 cm^{-1}, ~440 cm^{-1}, ~465 cm^{-1} and 520 cm^{-1} (Spectra are normalized to the A^2_g intensity.). (b) Optical microscopy images obtained in different laser exposure times within the same BP flake. (c) Laser exposure dependence of the intensity ratio between the A^2_g and Si peaks.

4. Practical applications as chemical sensors

Liquid-exfoliated BP was spin-coated on a field-effect transistor (FET) to investigate the charge transport characteristics. The drain current versus drain voltage curves in Fig. S4a show that the drain current modulation induced by the gate electric field is not exhibited. The reason why there is no field effect observed is due to the degradation of the BP FET, which is attributed to the introduction of charge carrier trap sites near the BP layer and the gate dielectric interface.

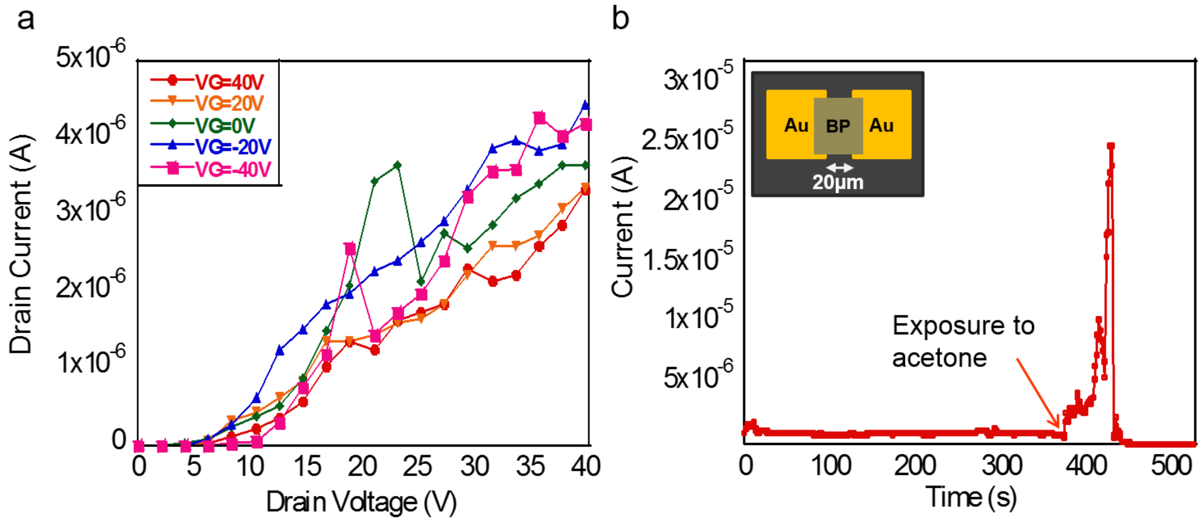

Fig. S4. (a) Drain current as a function of drain voltage of a liquid-exfoliated BP FET as the gate voltage varies from -40 V to 40 V. The BP solution was spin-coated at 1500 rpm for 60 s. (b) Current as a function of time in a liquid-exfoliated BP FET before and after the exposure to acetone. The drain voltage was fixed at 8 V.

The electrical sensitivity to acetone was tested by exposing acetone vapor to a liquid-exfoliated BP FET covered with a beaker. In the initial current-voltage (I-V) curve, the measured current was as small as 10^{-7}A, but the current was increased to 10^{-5}A right after the injection of acetone as shown in Fig. S4. This indicates that the device effectively detects acetone vapor. Previous study by Rahman and his coworkers suggested that adsorbed oxygen

during oxidation depletes electrons before the acetone injection, which results in a decrease in the concentration of mobile electrons in the liquid-exfoliated BP. In contrast, the current increase after the exposure to acetone is attributed to an increase in the electron concentration resulted from desorption of oxygen. However, understanding the sensing mechanism needs more work under controlled atmosphere. This sensing capability of the device demonstrates that liquid-exfoliated BP can be utilized in practical applications such as sensors.

Liquid-exfoliated BP thin films can be incorporated into a diode as a sensitizer in the reverse bias region in which the "off" current remains low. We fabricated a diode with a TiO_2 layer inserted between indium tin oxide (ITO) and liquid-exfoliated BP as shown in Fig. S5a. To prevent "short" between ITO and Al electrodes, the TiO_2 film was incorporated between ITO and BP layers. Fig. S5a shows the I-V curve of BP on TiO_2. At the reverse bias, the measured diode current was increased from approximately 8.5×10^{-5} A to1.4×10^{-4} A after exposure to acetone. This clearly shows the liquid-exfoliated BP acts as a sensitizer.

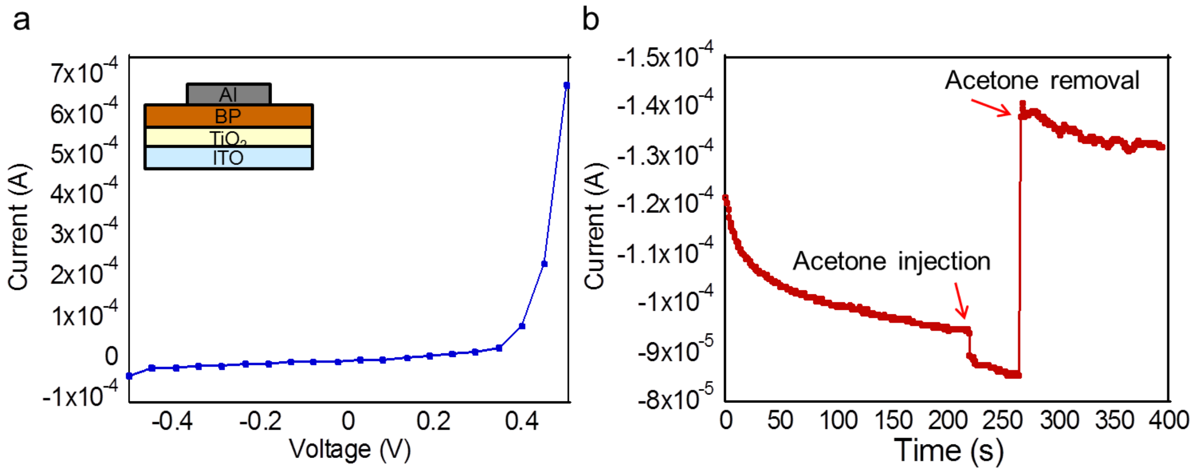

Fig. S5 (a) Current as a function of voltage of liquid-exfoliated BP/TiO_2. (b) Current as a function of time at V= - 0.3 V for a diode with TiO_2 layer inserted between ITO and liquid-exfoliated BP.
